# *In vitro* and *in vivo* efficacy, toxicity, bio-distribution and resistance selection of a novel antibacterial drug candidate

**DOI:** 10.1038/srep26077

**Published:** 2016-05-12

**Authors:** Jlenia Brunetti, Chiara Falciani, Giulia Roscia, Simona Pollini, Stefano Bindi, Silvia Scali, Unai Cossio Arrieta, Vanessa Gómez-Vallejo, Leila Quercini, Elisa Ibba, Marco Prato, Gian Maria Rossolini, Jordi Llop, Luisa Bracci, Alessandro Pini

**Affiliations:** 1 Department of Medical Biotechnologies, University of Siena, Siena, Italy; 2 SetLance srl, via Fiorentina 1, Siena, Italy; 3 Clinical Pathology Laboratory, Azienda Ospedaliera Universitaria Senese, Policlinico Le Scotte, viale Bracci, Siena, Italy; 4 Radiochemistry and Nuclear Imaging Group CIC biomaGUNE, San Sebastián, Spain; 5 Department of Experimental and Clinical Medicine, University of Florence, Italy; 6 Microbiology and Virology Unit, Florence Careggi University Hospital, Florence, Italy; 7 Don Carlo Gnocchi Foundation I.R.C.C.S., Florence, Italy

## Abstract

A synthetic antimicrobial peptide was identified as a possible candidate for the development of a new antibacterial drug. The peptide, SET-M33L, showed a MIC_90_ below 1.5 μM and 3 μM for *Pseudomonas aeruginosa* and *Klebsiella pneumoniae*, respectively. In *in vivo* models of *P. aeruginosa* infections, the peptide and its pegylated form (SET-M33L-PEG) enabled a survival percentage of 60–80% in sepsis and lung infections when injected twice i.v. at 5 mg/Kg, and completely healed skin infections when administered topically. Plasma clearance showed different kinetics for SET-M33L and SET-M33L-PEG, the latter having greater persistence two hours after injection. Bio-distribution in organs did not show significant differences in uptake of the two peptides. Unlike colistin, SET-M33L did not select resistant mutants in bacterial cultures and also proved non genotoxic and to have much lower *in vivo* toxicity than antimicrobial peptides already used in clinical practice. The characterizations reported here are part of a preclinical development plan that should bring the molecule to clinical trial in the next few years.

The emergence and dissemination of multidrug resistant (MDR) Gram-negative bacterial pathogens observed in recent years is a major challenge for antimicrobial chemotherapy, and because of its implications is now considered a major public health issue^1^. This “antibiotic resistance crisis” has been aggravated by the gap between the burden of infections due to MDR bacteria and the development of new antibiotics to tackle the problem^2^. The need for new antibiotics is urgent^3^.

Antimicrobial peptides (AMPs) are considered an interesting class of antibacterial molecules^4^. They have a positive net charge that allows them to interact selectively with anionic bacterial membranes and with other negatively charged structures, such as lipopolysaccharide (LPS), lipoteichoic acid (LTA) and DNA. Their mechanism of action is generally through specific binding to bacterial surfaces, by which they provoke cell permeation and in some cases inhibition of metabolic pathways. Unfortunately, two main problems have hindered the development of AMP-based drugs. The first is that the selectivity of natural antimicrobial peptides for bacteria is often low, so that they show a certain degree of toxicity for eukaryotic cells *in vitro*. The second is linked to the generally short half-life of peptides *in vivo*. These reasons make the way to market rather difficult for antimicrobial peptides^5^. Colistin and polymyxin B, two cyclic cationic lipodecapeptides binding bacterial LPS, discovered in the 1950s, are currently the only AMPs with antimicrobial activity available clinically for treatment of infections caused by MDR Gram-negative bacteria.

We previously identified a non-natural peptide sequence (KKIRVRLSA), particularly active against *P. aeruginosa,* with strong antimicrobial activity against a panel of Gram-negative bacteria. The peptide, called M33^6^; (hence SET-M33L), was obtained by optimization of previously reported peptides^7^. Its branched form^8^ made it more stable in biological fluids^9^,^10^,^11^,^12^,^13^,^14^. It acts by a two-step mechanism: 1) high affinity binding to LPS^15^; 2) disruption of bacterial membranes. SET-M33L was characterized for its biological activity against a number of Gram-negative MDR clinical isolates, including many cystic fibrosis isolates, as well as for its interaction with membranes, LPS and DNA, for its *in vitro* toxicity against several eukaryotic cell lines^16^, and for its hemolytic activity, lack of immunogenicity^15^ and ability to eradicate biofilms^17^. SET-M33L production includes a procedure to eliminate bio-products and is suitable for industrial development of a peptide-based drug^16^.

SET-M33L is currently undergoing preclinical development. Here we studied: i) antimicrobial activity against a large panel of MDR Gram-negative clinical isolates of *P. aeruginosa* and *K. pneumoniae*; ii) SET-M33L propensity to select for bacterial resistance, in comparison with colistin; iii) *in vivo* antibacterial activity of SET-M33L and its pegylated version SET-M33L-PEG^18^ in models of sepsis, pneumonia and skin infections caused by *P. aeruginosa*; iv) *in vivo* acute toxicity compared to the clinically available drug colistin; v) plasma clearance and biodistribution of radioiodinated SET-M33L and SET-M33L-PEG; vi) *in vivo* dose-response curve; vii) genotoxicity.

## Results

### Antimicrobial activity of SET-M33L against a large panel of *P. aeruginosa* and *K. pneumoniae* of clinical origin

Minimum inhibitory concentrations (MICs) of SET-M33L were determined against 76 clinical isolates of *Pseudomonas aeruginosa* and 73 clinical isolates of *Klebsiella pneumoniae*. The isolates were selected on the basis of their resistance phenotypes and major antibiotic resistance determinants, including resistance to colistin and carriage of carbapenemase-encoding genes (Table 1 and supplemental Tables S1 and S2).

Most of the *P. aeruginosa* strains (95%) showed a MIC in the range 0.7–1.4 μM (4–8 μg/mL) and a MIC_90_ of 1.4 μM (8 μg/mL). This value matched the modal MIC, which was independent of the different resistance phenotypes and resistance mechanisms (Table 1, supplemental Tables S1 and S2). *K. pneumoniae* strains on the whole showed similar MIC values, with most strains (90%) showing MIC values in the range 0.7–2.8 μM (4–16 μg/mL) and a MIC_90_ of 2.8 μM (16 μg/mL); no significant association between MIC values and resistance phenotypes/genotypes was observed except for colistin-resistant isolates, which on the whole had higher MIC values (range 2.8–>11.2 μM, 16–>64 μg/mL) (Table 1), as already observed for different cationic peptides with other major pathogens^19^,^20^. These findings sustain the hypothesis of an overlapping mechanism that confers resistance to both molecules in these strains, presumably also involving the same molecular target (i.e. LPS), as previously demonstrated^6^.

### Resistance selection

SET-M33L- and colistin-resistant mutant selection was attempted *in vitro* using the SET-M33L-susceptible (MIC 0.35 μM) and colistin-susceptible (MIC 0.15 μM) *K. pneumoniae* strain KKBO-1^21^. Tests were performed by plating cells on SET-M33L- or colistin-containing MHB. With this approach, colistin-resistant mutants were selected at a frequency of approximately 1 × 10^−7^ CFU, in line with previous results^21^, while no mutant strains was selected for SET-M33L using an inoculum up to 5 × 10^9^ CFU. Results of these experiments suggested a significantly lower SET-M33L propensity for resistance selection with respect to colistin (lower by a factor of at least 500).

### Therapeutic activity

#### Sepsis model

**Figure 1:**
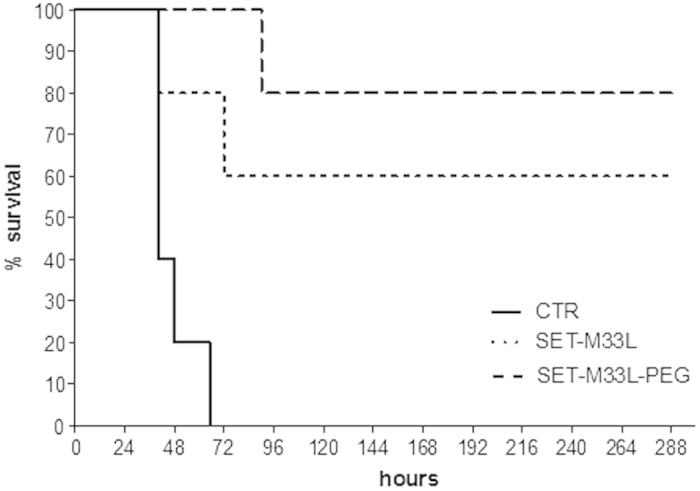
*In vivo* antibacterial activity of SET-M33L and SET-M33L-PEG peptides in sepsis animal model. 10 BALB/c neutropenic mice/group were injected i.p. with a lethal amount of *P. aeruginosa* PAO1 and then treated twice i.v. with SET-M33L or SET-M33-PEG (5 mg/Kg), 24 and 72 hours post-infection. Percentage survival (y-axis) is plotted as a function of time (x-axis); p < 0.02.

Neutropenic mice were injected i.p. with a lethal amount of *P. aeruginosa* PAO1 (1.5 × 10^3^ CFU/mouse). Then mice were treated twice i.v. with SET-M33L or SET-M33L-PEG (5 mg/Kg), 24 and 72 hours post-infection. Then animals were monitored for survival for 12 days. Survivals of 60% and 80% were obtained with SET-M33L or SET-M33L-PEG, respectively (Fig. 1). Note that this is a model of acute and rapidly progressive infection, since animals start to show signs of disease within 24 hours and controls died after 40–65 hours. Treatment with the peptides begun 24 h after onset of the infection. The relatively long interval between i.p. inoculation of bacteria and the start of i.v. treatment in animals with suppressed immune systems is a valid indication of the potency of the peptides *in vivo*.

#### Lung infection

**Figure 2:**
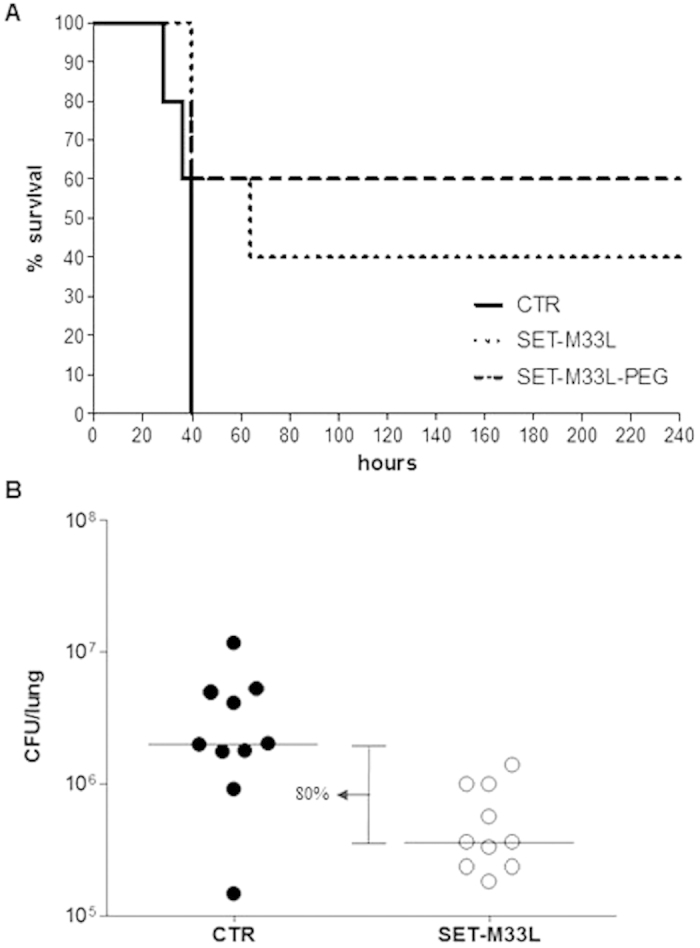
*In vivo* antibacterial activity of SET-M33L and SET-M33L-PEG peptides in lung infection. (**A**) Survival of neutropenic BALB/c mice after peptide treatment. All animals were injected i.t. with a lethal amount of *P. aeruginosa* PAO1. One group of animals was treated i.v. with 5 mg/Kg SET-M33L and one group with 5 mg/Kg SET-M33L-PEG, 1 and 16 hours post-infection. The control group (CTR) only received vehicle. The groups are indicated as described in the internal legend. Percentage survival (y-axis) is plotted as a function of time (x-axis); p < 0.05. (**B**) Scatter plots representing the CFUs/lung (y-axis) in treated and untreated non-neutropenic mice (each circle corresponds to one mice). All animals were injected i.t. with *P. aeruginosa* PAO1. One group was treated i.t. with a single 5 mg/Kg dose of SET-M33L (white circles). The control group only received vehicle (black circles). The horizontal lines represent the median and the difference between medians is indicated as a percentage. p = 0.05. There were 10 mice/group.

Neutropenic mice were injected intra-tracheally (i.t.) with a lethal amount of *P. aeruginosa* PAO1 (1.5 × 10^3^ CFU/mouse). They were then treated twice i.v. 1 and 16 hours post-infection with SET-M33L or SET-M33L-PEG 5 mg/Kg and monitored for survival for 10 days. We obtained 40% and 60% survival with SET-M33L and SET-M33L-PEG, respectively (Fig. 2A).

In a different model, non-neutropenic mice were first infected i.t. with *P. aeruginosa* PAO1 (1 × 10^6^ CFU/mouse) and then treated once i.t. with SET-M33L 5 mg/Kg. Animals were sacrificed 12 hours after treatment and the lungs collected for CFU count. We observed an 80% reduction in CFU in lungs of animals treated with SET-M33L (Fig. 2B), confirming the strong activity of the peptide also when administered locally in the lungs, thus mimicking the aerosol administration.

#### Skin infection

**Figure 3:**
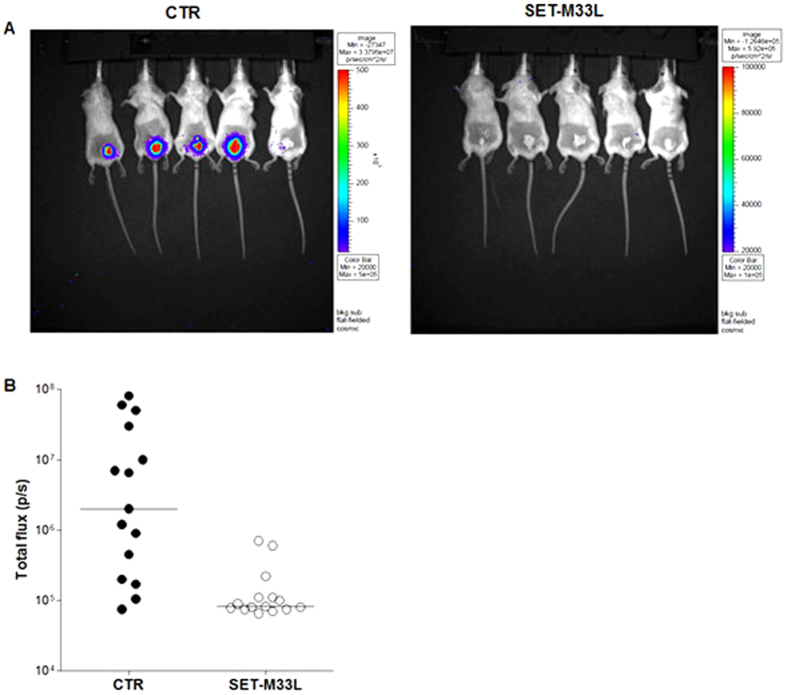
*In vivo* antibacterial activity of SET-M33L peptide in skin infection. 15 neutropenic mice per group (BALB/c) were infected on abraded skin with *P. aeruginosa* P1242 and then treated every day with 10 mg/ml SET-M33L-lotion (SET-M33L) or with SET-M33L free-lotion (CTR). (**A**) Example of images of five animals at day 2. (**B**) Scatter plots representing the photon flux per second (p/s) emitted by bacteria at day 2 from all mice (each circle corresponds to one animal). The horizontal lines indicate the median value. p < 0.03.

Neutropenic mice were infected on abraded skin with *P. aeruginosa* P1242 (5 × 10^3^ CFU/mouse), a modified strain expressing the luciferase gene and its substrate. 50 μl of a M33- based lotion (10 mg/ml) was spread on the infection site every day. One day post-infection the treatment produced a significant reduction in *P. aeruginosa* load as indicated by a significant decrease in the total photon flux emitted by bacteria. This protective effect was especially evident 2 days after the challenge (Fig. 3). The infection healed spontaneously and completely 7 days after infection (not shown).

### Acute toxicity

**Figure 4:**
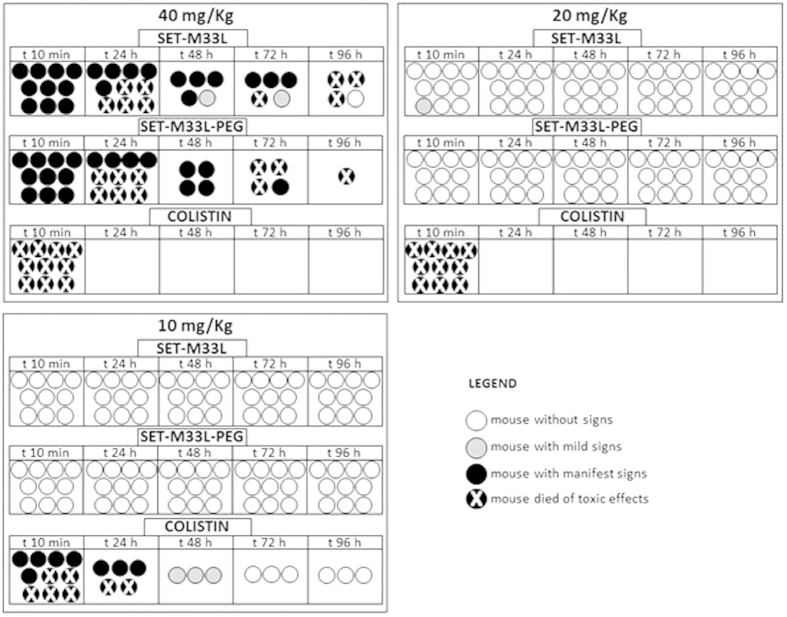
Acute *in vivo* toxicity of SET-M33L, SET-M33L-PEG and colistin at 40 mg/Kg, 20 mg/Kg and 10 mg/Kg, given in a single dose. Ten mice/group (each circle represents one mouse) were inoculated i.v. with SET-M33L, SET-M33L-PEG or colistin and were monitored for 96 hours. Different scales of grey indicate severity of signs as described in the legend. Toxicity scores were rated as described in Material and Methods.

As preliminary toxicity evaluation we first compared the acute toxicity of SET-M33L and SET-M33L-PEG with that of colistin. Animals (10 BALB/c mice/group) were injected i.v. with different amounts of SET-M33L, SET-M33L-PEG or colistin, in single dose, in the range 5–40 mg/kg (Fig. 4), and were monitored for 4 days after inoculation of the peptide. SET-M33L and SET-M33L-PEG were not lethal and no signs of toxicity were observed up to 20 mg/kg. At 40 mg/Kg SET-M33L and SET-M33L-PEG produced similar toxicity profiles with strong signs of toxicity and 90–100% mortality after 96 hours. Colistin produced strong signs of toxicity already at 10 mg/Kg: 50% mortality immediately after inoculation and 70% mortality after 24 hours. Colistin induced sudden death in 100% of mice at 20 and 40 mg/Kg.

Animal weight was monitored every day. All animals treated with 40 and 20 mg/Kg showed less than 5% weight loss after 24 h. 48 h after the challenge they regained initial weight. No weight variation was recorded in animals treated with 10 mg/Kg of the compounds (not shown).

### Biodistribution and plasma clearance

For biodistribution and clearance, SET-M33L and SET-M33L-PEG were synthesized with a single additional tyrosine at the peptide C-terminal. Since SET-M33L is a branched peptide with the branching core at the C-terminus, the additional tyrosine was distant from the active peptide sequence, i.e. pharmacophore.

**Figure 5:**
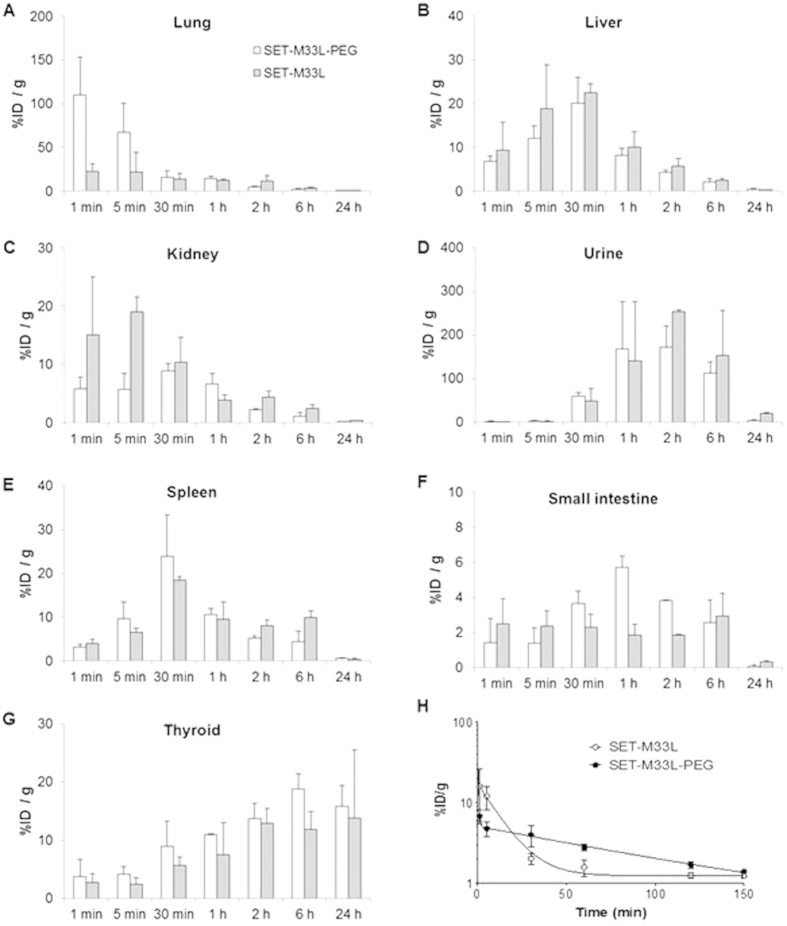
Accumulation of [^125^I]SET-M33L and [^125^I]SET-M33L-PEG, expressed as % of injected dose per gram of tissue (%ID/g) into the different organs and plasma after intravenous administration and organ dissection or plasma collection. (**A**–**G**) Peptide bio-distribution in organs indicated above each panel. The Y axis scale is different in every graph. (**H**) Plasma clearance. Fitted curves are exponential for SET-M33L and bi-exponential for SET-M33L-PEG. The graph was obtained using GraphPad Prism software.

Peptides were labeled with ^125^I for *ex vivo* radioactivity measurement and gamma count after organ dissection (Fig. 5), and with ^124^I for *in vivo* positron emission tomography (PET) analysis (Figures S1 and S2).

With *ex vivo*^125^I measurement, high early accumulation of SET-M33L-PEG was observed in the lungs (109.9 ± 43% ID/g at 1 min), followed by elimination in one hour (14.1 ± 2.8% at 1 hour). Accumulation of peptide SET-M33L in the lungs was lower, with values of %ID/g below 25% throughout the study (Fig. 5A). Time-activity-curves in the liver followed a similar profile for both peptides, with a progressive increase up to 30 minutes after administration (20.1 ± 6% and 22.5 ± 2% for SET-M33L-PEG and SET-M33L at 30 min, respectively) and a progressive decrease afterwards. Residual activity in the liver was negligible by 24 hours (Fig. 5B). Radioactivity in the kidneys (Fig. 5C) from both peptides suggested elimination mainly via urine, and this was confirmed by the increase in radioactivity in urine at t > 30 min and by the simultaneous reduction in radioactivity in kidneys (Fig. 5D). Kidney accumulation was, however, faster for SET-M33L, in line with plasma clearance described below. Significant amounts of radioactivity from both peptides were also detected in the spleen, with greater persistence of SET-M33L after 6 hours (Fig. 5E). Accumulation of radioactivity was recorded in the small intestine. The profiles were different for the two peptides. For SET-M33L-PEG, accumulation peaked at 1 hour (5.7 ± 2.6% ID/g) and slowly decreased afterwards. For SET-M33L, steady accumulation was observed up to 6 hours, with values of %ID/g in the range 1.5–2.5% (Fig. 5F). The longer increase in radioactivity in the thyroid gland suggested metabolization of the labeled peptides and subsequent release of ^125^I. %ID/g was 16.8 ± 3.6% and 13.8 ± 11.7% for SET-M33L-PEG and SET-M33L, respectively, at 24 hours (Fig. 5G).

Accumulation of radioactivity in testicles and brain was not significant throughout the study with %ID/g < 1% at all time points (not shown).

Time activity curves in plasma showed different behavior for the two peptides (Fig. 5H). For SET-M33L-PEG, there was a rapid initial distribution phase followed by a slower log-linear decrease in concentration. Mean plasma clearance was calculated as 79.45 min. For SET-M33L, the initial distribution phase could not be identified and the plot fitted a single exponential curve; mean plasma clearance was 8.6 min.

PET data obtained with ^124^I-labeled peptides (Figure S1) gave similar results to those obtained with ^125^I peptides, with the expected discrepancies. For example, lower radioactivity was recorded in the lungs. These differences can be explained by the fact that *in vivo* (^124^I), the results were expressed as %ID/cm^3^, whereas *ex vivo* (^125^I) they were expressed as %ID/g. This data can be compared when tissue density is close to 1 g/cm^3^, which is not the case for a functional (breathing) lung. Illustrative PET images are included in the supplementary material (Figure S2).

### Dose-response curve

**Figure 6:**
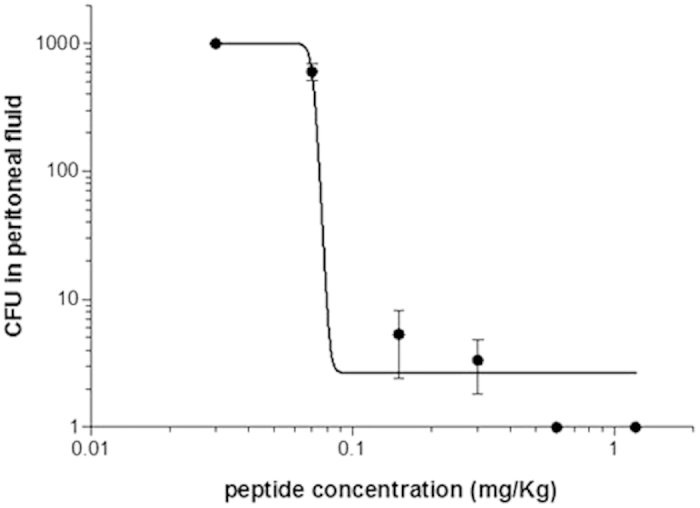
*In vivo* dose-response activity of SET-M33L. 5 BALB/c mice per group were injected i.p. with 1 × 10^7^ CFU/mouse of *P. aeruginosa* PAO1 and after 15 minutes the antimicrobial peptide SET-M33L was inoculated i.p. at different doses (0.03–1.2 mg/Kg). Five hours after bacterial challenge, peritoneal fluid was analyzed for bacteria and CFU count (y-axis). The scales of the axes are logarithmic.

Dose-response activity was evaluated for SET-M33L in a mouse model infected with *P. aeruginosa* PAO1. Animals were infected i.p. with bacteria (1 × 10^7^ CFU/mouse), and treated i.p. with scalar amounts of SET-M33L 15 min post-infection. After 5 hours, blood and peritoneal fluid were analyzed for bacteria. Only peritoneal fluid gave reliable results (Fig. 6). ED50 for SET-M33L was 0.078 mg/Kg.

### Gene toxicity

Gene toxicity was assessed by testing the potential of SET-M33L to induce micronuclei in human lymphocytes. Human lymphocytes in whole blood culture, stimulated to divide by addition of PHA 48 hours prior to treatment, were exposed to SET-M33L for 3 hours with and without exogenous metabolic activation (S9 mix), and for 20 hours without S9 mix. In preliminary experiments (not shown), the maximum final concentration to which the cells were exposed was 500 μg/ml; the purpose was to test to the maximum concentration as recommended in the guidelines for this assay.

SET-M33L did not cause any statistically significant increase in the number of binucleate cells containing micronuclei with respect to the vehicle controls in the 3-hour or the 20-hour test (Table 2). The positive control compounds caused statistically significant increases in the number of binucleate cells containing micronuclei under appropriate conditions, demonstrating the efficacy of the S9 mix and the sensitivity of the test system.

## Discussion

The standard regulatory procedures indicated by the European Medicines Agency, Committee for Medicinal Products for Human Use, specify that before a new therapeutic entity, also called investigational medicinal product (IMP), can be given to humans, researchers and developers must first test it thoroughly in animals. The main aims of these pre-clinical studies are: i) IMP efficacy and toxicity; ii) IMP pharmacokinetics; iii) IMP formulation, for example a capsule or injection, suitable for early studies in humans.

The synthetic antimicrobial peptide SET-M33L is currently undergoing preclinical development and continued experimentation was planned to meet international requirements. Here we report its activity *in vivo,* using models of infections to reproduce common human infections due to *P. aeruginosa*, such as those provoked by abdominal trauma^22^,^23^, or highly diffused in cystic fibrosis patients^24^, or affecting skin wounds and burns^25^. Antimicrobials against *P. aeruginosa* are urgently needed^26^ because the bacterium is a major cause of infections in humans, and its clinical interest is dramatically increasing due to its growing multi-resistance to traditional antibiotics^27^.

In a preliminary study^6^ we tested SET-M33L in sepsis models where SET-M33L and bacteria were administered i.p. to animals with active immune systems. In order to reduce the contribution of individual native and adaptive immune response to infection we decided to use neutropenic animals. In the models reported here, the immune system of mice was impaired with cyclophosphamide in the first 3–4 days post-infection. Recovery from infection obtained in our models was therefore almost completely due to the antibacterial peptides administered. These models are much more indicative of peptide activity *in vivo* and strong therapeutic activity was evident in all models. The peptide SET-M33L and its pegylated form were tested by administering the molecule in doses compatible with clinical use and by routes (i.v., i.t. or topical) already used for antimicrobial peptides in humans.

The preliminary toxicity *in vivo* was particularly encouraging since SET-M33L proved to be much less toxic than colistin. Colistin is a peptide antibiotic, like SET-M33L. It is extensively used in clinical practice^28^,^29^,^30^ at the doses we tested here for SET-M33L^31^,^32^. The results of peptide acute toxicity testing, along with *in vivo* experiments of therapeutic and dose-response activity, suggest that the selectivity of SET-M33L for bacteria will provide an acceptable therapeutic index.

The pharmacokinetic and biodistribution analyses showed that both peptides were eliminated mainly via urine, although metabolization by the liver cannot be excluded. The latter statement is mainly true for SET-M33L-PEG, which shows higher accumulation in the small intestine after 1–2 hours, in line with its longer plasma clearance.

Unlike SET-M33L, SET-M33L-PEG accumulated in lungs in the first minutes after administration; subsequently the two peptides showed similar profiles in lungs. This different behavior between the two peptides was perfectly reproduced *ex vivo* and *in vivo* (PET). Importantly, the peptides did not accumulate significantly in the brain or testicles.

Plasma clearance showed longer persistence in circulation of SET-M33-PEG than SET-M33L (79.45 min and 8.6 min, respectively), and this may partially explain the better performance of the pegylated molecule in eradicating infections *in vivo*.

A crucial aspect emerging from this study is the very low propensity of SET-M33L to select resistant strains, compared to colistin, which proved at least 500 times more prone to select resistant mutants in the same experimental conditions. At present, the available information on mutant selection frequencies with colistin is overall scarce and data regarding mutants selection for AMPs are lacking. The mutant selection frequency towards colistin, here reported for the KKBO-1 strain, was overall similar to that previously observed for the same strain^21^. Nevertheless, remarkably variable mutation frequencies for colistin were observed among different *K. pneumoniae* strains^33^ and variability among mutation frequencies for SET-M33L with different strains could not be excluded.

Finally, SET-M33L proved not to be genotoxic for eukaryotic cells. This issue is not trivial, considering the propensity of this molecule to strongly bind DNA^15^, evidently due to an excess of positive charges.

All the characterizations described in this study make the antimicrobial peptide SET-M33L a good candidate to become a new antibacterial agent for the treatment of severe infections due to MDR pathogens, and especially indicated against *P. aeruginosa and K. pneumoniae*. The preclinical development plan, including safety pharmacology in higher animals, bio-analytical manufacturing methods and formulation, is expected to conclude within two years, so that clinical trials can begin in two or three years.

## Materials and Methods

### Peptide synthesis

#### General settings

All peptides were produced in tetra-branched form with an automated synthesizer (Syro, MultiSynTech, Witten, Germany) on a solid support using standard Fmoc chemistry. Resins and protected amino acids were purchased from Iris Biotech. Side chain protecting groups of amino acids were 2,2,4,6,7-pentamethyldihydro benzofuran-5-sulfonyl for R, t-butoxycarbonyl for K and t-butyl for S. The final peptides were cleaved from the solid support and deprotected in one step by treatment with TFA containing triisopropylsilane and water (95/2.5/2.5), and precipitated with diethyl ether. Crude peptides were purified by reversed-phase chromatography on an XBridge Peptide BEH C18 300 Å 10 μm 250 × 19 mm column (Waters), using 0.1% TFA/water as eluent A and acetonitrile as eluent B, and a linear gradient from 83% to 70% A. Purified peptides were obtained as trifluoroacetate salts (TFacetate). The exchange from TFacetate to acetate form was carried out using a quaternary ammonium resin in acetate form (AG1-X8, 100–200 mesh, 1.2 meq/ml capacity, Biorad). The resin to peptide ratio was 2000:1. Resin and peptide were stirred for 1 h, the resin filtered off, washed extensively and the peptide recovered and freeze-dried^16^. Final peptide purity (always >95%) and identity was confirmed by reversed phase chromatography on a Phenomenex Jupiter C18 analytical column (300 Å, 5 μm, 250 × 4.6 mm) and by mass spectrometry MALDI ToF/ToF (Ultraflex III, Brucker Daltonics, Bremen Germany).

#### SET-M33L, SET-M33L-PEG, SET-M33L-Tyr and SET-M33L-PEG-Tyr

SET-M33L was produced on a Fmoc4-Lys2-Lys-b-Ala Wang resin. SET-M33L-PEG, SET-M33L-Tyr and SET-M33L-PEG-Tyr were synthesized on TentaGel S RAM resin. For SET-M33L-PEG, Fmoc-NH-PEG(4)-COOH was used as first coupling step. For SET-M33L-Tyr and SET-M33L-PEG-Tyr, Fmoc-Tyr(tBu)-OH was used in the first coupling step and Fmoc-βAla-OH and Fmoc-NH-PEG(4)-OH were used for the second coupling step of SET-M33L-Tyr and SET-M33L-PEG-Tyr, respectively. Two consecutive couplings of Fmoc-Lys(Fmoc)-OH were used for all peptides to build the branched core.

### MIC 90 and 50

MIC values were determined against a large collection of clinical isolates. Most isolates were collected during routine susceptibility testing procedures at the Clinical Microbiology Unit of Careggi University Hospital in Florence, while specific isolates were selected according to their phenotype or genotype (e.g. carriage of carbapenemase-encoding genes, inclusion in epidemic bacterial clones, resistance to colistin) from a collection of isolates already available at Department of Medical Biotechnologies, University of Siena. Detailed features of each strain, including relevant resistance phenotypes and genotypes were reported in supplemental Tables S1 and S2. MICs were determined in triplicate using a standard microdilution assay according to the guidelines of the Clinical and Laboratory Standards Institute^34^. SET-M33L was assayed using concentrations in the range 0.09–11.2 μM (0.5–64 μg/mL); testing was performed using a bacterial inoculum of 5 × 10^4^ CFU/well prepared in cation-supplemented Mueller-Hinton broth (MHB) (Becton Dickinson, Franklin Lakes, NJ, USA) in a final volume of 100 μl. Results were recorded after 18–20 h of incubation at 35 °C.

### Bacterial resistance

Selection was performed on an MHB-based selection medium containing 1% low electro-osmosis agarose as solidifying agent and 11.2 μM SET-M33L (64 μg/mL). Agarose was preferred to agar as solidifying agent due to the observed loss of antimicrobial activity of SET-M33L in selection media solidified with the latter, possibly due to the charged nature of agar. *Klebsiella pneumoniae* KKBO-1 strain was grown in MHB at 37 °C to OD600 0.35 and up to 5 × 10^9^ colony forming units (CFU) were spread on Petri dishes containing 20 ml of the selection medium. Plates were incubated for 16–18 h at 37 °C. Colistin-containing selection medium (equimolar concentration with respect to SET-M33L) was used as control for the selection of colistin-resistant mutants. Experiments were performed in triplicate.

### Animal models: sepsis, lung infection, skin infection

Animal procedures were approved by the Ethics Committees of the Azienda Ospedaliera Universitaria Senese on November 18, 2010, and of the Italian Ministry of Health on September 21, 2012. Eight-week-old (20 g) BALB/c female mice (Charles River) were used in all experiments. The animals were maintained and handled in accordance with the Guidelines for Accommodation and Care of Animals (European Convention for the Protection of Vertebrate Animals Used for Experimental and Other Scientific Purposes) and internal guidelines. For each experiment the animals were housed in groups of five mice per cage upon arrival and maintained with food and water ad libitum for 5 days before the beginning of treatment. All p values were calculated using GraphPad Prism 5.0 software.

#### Sepsis model

In the *Pseudomonas* model of sepsis^35^ animals were rendered neutropenic by i.p. administration of cyclophosphamide (Sigma-Aldrich C7397) at 150 mg/kg (300 μl of a 10 mg/ml solution) 4 days and 1 day before infection. Sepsis was produced infecting animals i.p. with a lethal amount of *P. aeruginosa* PAO1 (1.5 × 10^3^ CFU/mouse) mixed in 500 μl PBS. After 24 and 72 h, the peptide was inoculated i.v. in the caudal vein as 0.2 ml PBS solution containing 5 mg/kg of SET-M33L or SET-M33L-PEG. Control animals only received PBS. Surviving mice were monitored for 12 days. Moribund animals were killed humanely to avoid unnecessary distress. Groups consisted of 10 animals.

#### Lung Infection models

We set up two lung infection models, one for i.v. and the other for intra-tracheal (i.t.) treatment. In the i.v. model, animals were made neutropenic as described in the sepsis model. For both models, on the day of infection, animals were first anesthetized with Avertin (Sigma-Aldrich 2,2,2-tribromoethanol T48402) (600 μl i.p. of 10 mg/ml solution). Ten minutes later, bacteria (*P. aeruginosa* PAO1, 1–3 × 10^3^ CFU/mouse for i.v. treatment, and 1 × 10^6^ CFU/mouse for i.t. treatment) were instilled i.t. through a small incision in the trachea where a 22-G catheter connected to a syringe was inserted (volume injected 50 μl)^35^. In the i.v. treatment model the wound was closed with a small clip. One hour and 16 h after bacterial administration the peptide was inoculated in the caudal vein with 0.2 ml PBS solution containing 5 mg/kg of SET-M33L or SET-M33L-PEG. Control animals received only PBS. Surviving mice were monitored for 10 days. Moribund animals were killed humanely to avoid unnecessary distress. Groups consisted of 10 animals.

Immunocompetent mice were used in the i.t. treatment model. A few minutes after inoculation of bacteria, 30 μl of PBS solution containing SET-M33L (5 mg/kg) was delivered to the lungs through the same tracheal catheter with a different syringe. The wound was then closed with a small clip. After 12 hours the animals were sacrificed and the lungs collected and homogenized in 500 μl of sterile solution (PBS-Triton X 0.1%) using a sterile glass Potter homogenizer. Samples were diluted serially and 100 μl of each dilution was spread in duplicate on appropriate agar plates for colony count.

#### Skin infection

Mice were immunosuppressed by i.p. injection of cyclophosphamide (150 mg/kg) 3 days before infection. On the day of infection, mice were anesthetized i.p. with ketamine (80 mg/kg) and xilazine (5 mg/kg), then an area of 4 cm^2^ on their backs was shaved of all fur and the skin was abraded with sandpaper until it glistened. 20 μl suspension containing *P. aeruginosa* P1242 (5 × 10^3^ CFU/mouse) was deposited on the abraded area. This bacterium expressed the luciferase gene and luciferin substrate under control of a constitutive P1 integron promoter^36^. SET-M33L peptide was mixed with a commercial cream (Essex, Schering-Plough) obtaining a 10 mg/ml concentrated lotion of SET-M33L, that was applied topically to the infected area of 15 mice once a day after infection in an approximate volume of 50 μl. The course of the infection was monitored every day for 7 days post-infection by imaging the animal dorsal side up in an IVIS-200 imaging system (Xenogen Corporation, Alameda, CA) after anesthetization with 2.5% isoflurane. Total photon emission from defined areas of the images of each mouse was quantified with the Living Image software package.

### Acute toxicity

Mice were treated by a single i.v. administration of different amounts (40, 20, 10, 5 mg/Kg) of SET-M33L, SET-M33L-PEG or colistin sulfate (Sigma-Aldrich, C4461). Signs of toxicity were checked four times a day by visual inspection. A toxicity score was assigned for the following signs: wiry coat and poor motility = mild signs; very wiry coat, abundant lachrymation and poor motility even under stimulation = manifest signs. Animals were monitored for 4 days after inoculation of the peptide. Mice were weighed every day from arrival to the last day of the experiment. Moribund animals were killed humanely to avoid unnecessary distress.

### Radioiodination and biodistribution studies

#### Animals

Biodistribution studies with radioiodinated peptides were performed at CIC biomaGUNE using male BALB/cJRj mice (9 weeks, Janvier). The animals were maintained and handled in accordance with the Guidelines for Accommodation and Care of Animals (European Convention for the Protection of Vertebrate Animals Used for Experimental and Other Scientific Purposes) and internal guidelines. All experimental procedures were approved by the Ethical Committee of CIC biomaGUNE and by the Departamento de Promoción Económica, Medio Rural y Equilibrio Territorial de la Diputación Foral de Guipúzcoa.

#### Radiolabeling of the peptides

The radioiodination of the peptides was carried out by electrophilic aromatic substitution on the tyrosine residues. To do so, a solution of the peptide (50 μg/50 μL) was incubated with Na[^125^I]I (Perkin Elmer, 37 MBq) or Na[^124^I]I (Perkin Elmer, 74 MBq) in phosphate buffered saline (100 μl, pH 7.4) for 30 min at 25 °C in the presence of Iodobeads^®^. The reaction crude was purified by retention in a C-18 cartridge (Sep-Pak^®^ Light, Waters) and subsequent elution using 0.1% aqueous TFA solution/ethanol 20/80 (1 ml). The solvent was evaporated and the residue reconstituted with ethanol/water (10%, 100 μl). Chemical and radiochemical purity were determined by radio-HPLC, using a Mediterranean Sea 18 column (4.6 × 150 mm, 5 μm) as the stationary phase and 0.1% solution of TFA in water (A) and 0.1% solution of TFA in methanol (B) as mobile phase. The following gradient was used; initial: A-60% B-40%; 4 min: A-60% B-40%; 14 min: A-20% B-80%; 18 min: A-60% B-40%; 20 min: A-60% B-40%. Injected volume was 20 μl. Retention time for the two peptides was 8.3 min in both cases.

Radiochemical stability of the radiolabelled peptides was determined *in vitro* in different media: (i) Phosphate buffered solution, and (ii) 0.1% solution of TFA in water**/**0.1% solution of TFA in methanol (20/80). To do so, the peptides were incubated at 37 °C in the corresponding media; samples were withdrawn at different times (1, 2, 4, 6, 48 hours) and analyzed by radio-HPLC using the conditions described for quality control.

#### PET studies

PET experiments (n = 3 per peptide) were performed using an eXploreVista-CT small animal PET-CT system (GE Healthcare). Anesthesia was induced with 3% isoflurane and maintained with 1.5–2% of isoflurane in 100% O_2_. The corresponding ^124^I-labeled peptide (2.7 ± 0.7 MBq, 150 μl) was injected into a lateral tail vein concomitantly with the start of a PET dynamic acquisition (energy window: 400–700 KeV). Two beds were defined to acquire whole body images (frames: 4 × 20 s, 4 × 60 s, 4 × 2 min, 4 × 4 min, 5 × 10 min, 5 × 20 min total acquisition time = 179 min). After acquisition, a CT scan (X-Ray energy: 40 kV, intensity: 140 μA) was performed for subsequent attenuation correction of the reconstructed image. Random and scatter corrections were also applied to the reconstructed image (2DOSEM iterative algorithm, 4 iterations).

PET-CT images of the same animal were co-recorded and analyzed using the PMOD image processing tool. Volumes of interest (VOIs) were centered on major organs and time-activity curves, expressed as percentage of injected dose per cm^3^ of tissue, were plotted.

#### *Ex-vivo* biodistribution: dissection and gamma counting

Animals (3 mice per compound and time point) were anesthetized with isoflurane and a saline solution of ^125^I-labelled peptide (1.8 ± 0.5 MBq/150 μl) was injected through a lateral tail vein. At pre-determined times, animals were sacrificed by perfusion using saline solution. Major organs were quickly removed, rinsed twice with deionized water and measured in an automatic gamma counter (2470 Wizard, PerkinElmer). Blood and urine samples (0.3 ml) were also obtained just before perfusion and blood was processed to separate the plasma. Plasma, blood and urine were also counted in the gamma counter.

To determine the half-life of both peptides in blood, time-activity curves were modeled with mono- (SET-M33L) or bi-exponential (SET-M33L-PEG) functions using GraphPad Prism 6 (GraphPad Software, Inc.).

### Dose-response curve

BALB/c mice (20 g ± 1) were injected i.p. with 1 × 10^7^ CFU/mouse of *P. aeruginosa* PAO1 in 500 μl PBS. Fifteen minutes after bacterial administration, the antimicrobial peptide SET-M33L was inoculated i.p. at different concentrations: 0.03 mg/Kg, 0.07 mg/Kg, 0.15 mg/Kg, 0.3 mg/Kg, 0.6 mg/Kg, 1.2 mg/Kg. Control animals only received PBS. Each group consisted of 5 animals (only 3 were used to plot the graph, see below). Five hours after bacterial infection, the animals were sacrificed and peritoneal fluid was obtained by washing with 5 ml PBS. Samples were diluted serially and 100 μl of each dilution was spread on appropriate agar plates for colony count.

The results were processed using GraphPad Prism 5.0 software. The graph was plotted with only three mice per group because the plates with the highest and lowest number of CFUs in each group were excluded.

### *In vitro* micronucleus test in human lymphocytes

Analysis of micronucleated cells was based on the criteria described by Fenech and Morley^37^ and Fenech^38^. The study described below was conducted in compliance with the following Good Laboratory Practice standards: The UK Good Laboratory Practice Regulations (Statutory Instrument 1999 No. 3106, as amended by Statutory Instrument 2004 No. 994); OECD Principles of Good Laboratory Practice (as revised in 1997), ENV/MC/CHEM (98) 17; EC Commission Directive 2004/10/EC of 11 February 2004 (Official Journal No. L 50/44). Human lymphocytes from informed donors were pooled and diluted with HML media (RPMI 1640, supplemented with 10% fetal calf serum, 0.2 IU/ml sodium heparin, 20 IU/ml penicillin, 20 μg/ml streptomycin and 2.0 mM L-glutamine). As lymphocytes do not normally undergo cell division, they were stimulated to do so by addition of phytohaemagglutinin (PHA), a naturally occurring mitogen. Cultures were established from the prepared (pooled) sample and dispensed as 5 ml aliquots (in sterile universal containers) so that each culture contained blood (0.4 ml), HML media (4.5 ml) and PHA solution (0.1 ml). The cultures were then incubated at 37 °C, and the cells were resuspended (twice daily) by gentle inversion.

Lymphocyte cultures were incubated for approximately 48 hours after stimulation with PHA, before addition of SET-M33L; controls were appropriately diluted with vehicle. S9 homogenate was present in appropriate cultures at a final concentration of 2% v/v. Before treatment, all cultures were centrifuged and resuspended in the required volume of fresh medium, taking treatment volume and S9 mix volume into account. SET-M33L preparations were added to cultures at 10% v/v. Cultures were incubated at 37 °C for 3 or 20 hours.

The cells were centrifuged and the medium replaced with fresh medium. Cytochalasin B, at a final concentration of 6 μg/ml, was then added to all cultures. The cultures were incubated for a further 17 hours until the scheduled harvest time.

The cells were harvested by centrifugation at 500 g for 5 minutes. The supernatant was removed and the cell pellet re-suspended and treated with 4 ml of hypotonic solution (0.075 M KCl) at 37 °C. The cultures were incubated for 3 minutes at 37 °C to cause swelling. They were then shaken and after slowly pouring 4 ml of ice-cold fixative (3:1 v/v methanol:acetic acid) onto the culture surface, they were slowly inverted to mix. The cultures were centrifuged at 500 g for five minutes. The supernatant was removed and the cell pellet re-suspended. A further 4 ml fresh fixative was then added and the cells were stored at about 4 °C until slide preparation. The cultures were centrifuged at 500 g for 5 minutes and the supernatant removed. Pre-cleaned microscope slides were prepared for each culture by aliquoting resuspended cells onto the slides, and allowing the slides to air-dry. Two slides were prepared from each culture. The prepared slides were examined by fluorescence microscopy. The incidences of mononucleate, binucleate and polynucleate cells were assessed per culture per 1000 binucleate cells. The occurrence of unusual numbers of cells undergoing mitosis, polyploid cells, necrotic cells or debris, for example, was also noted.

SET-M33L concentrations were selected for evaluation in a preliminary test. The highest concentration was taken to be the one that depressed the cytokinesis-block proliferative index (CBPI) equivalent to 55 ± 5% cytotoxicity with respect to the concurrent vehicle control or, where no cytotoxicity was observed. Prior to micronucleus analysis, all slides were randomly coded.

Positive controls were: cyclophosphamide: 10 μg/mL (3 h, +S9 mix); mitomycin C: 0.2 μg/mL (3 h, −S9 mix); 0.05 μg/mL (20 h, −S9 mix); colchicine: 0.06 μg/mL (3 h, −S9 mix); 0.01 μg/mL (20 h, −S9 mix).

## Additional Information

**How to cite this article**: Brunetti, J. *et al*. *In vitro* and *in vivo* efficacy, toxicity, bio-distribution and resistance selection of a novel antibacterial drug candidate. *Sci. Rep.* **6**, 26077; doi: 10.1038/srep26077 (2016).

## Supplementary Material

Supplementary Information

## Figures and Tables

**Table 1:** Distribution of MIC values of SET-M33L in the collection of clinical bacterial isolates, including distribution with respect to resistance phenotypes.

Species and resistance phenotypes (no. isolates)^a^^,^^b^	MIC								
	[μM]	0.18	0.35	0.7	1.4	2.8	5.6	11.2	>11.2
	[μg/mL]	1	2	4	8	16	32	64	>64
*Pseudomonas aeruginosa* (76)		0	1	8	64	2	0	1	0
MDR (32)		0	0	2	29	1	0	0	0
MDR, MBL (11)		0	0	0	11	0	0	0	0
non-MDR (44)		0	1	6	35	1	0	1	0
*Klebsiella pneumoniae* (73)		0	0	22	33	11	3	3	1
MDR (31)		0	0	12	10	5	1	2	1
MDR, CP, CR (6)		0	0	0	0	2	1	2	1
MDR, CP (10)		0	0	6	2	2	0	0	0
MDR, ESBL, CR (1)		0	0	0	0	1	0	0	0
MDR, ESBL (12)		0	0	4	8	0	0	0	0
non-MDR (42)		0	0	10	23	6	2	1	0
Non-MDR, ESBL (2)		0	0	0	2	0	0	0	0
	**MIC**_**50**_	**[μM]**	**[μg/mL]**		**MIC**_**90**_	**[μM]**	**[μg/mL]**		
*Pseudomonas aeruginosa* (no. 76)		1.4	8			1.4	8		
*Klebsiella pneumoniae* (no. 73)		1.4	8			2.8	16		

^a^Strains were identified according to the presence of relevant resistance phenotypes and genotypes as follows: MDR, multidrug resistant (according to the definition of Magiorakos *et al*.^39^); CP, carbapenemase-producing; CR, colistin-resistant; ESBL, extended spectrum β-lactamase-producing; MBL, metallo-β-lactamase-producing.

^b^Bacterial strains belonging to the successful epidemic *P. aeruginosa* Sequence Type 621, producing the IMP-13 MBL, and *K. pneumoniae* Sequence Type 512, producing a KPC-type carbapenemase, were included.

**Table 2:** Micronucleus tests with SET-M33L peptide in human lymphocytes.

Compound/concentration (μg/ml)	Mean of binucleate cells containing micronuclei per 1000 cells		
	3h − S9	3h + S9	20h − S9
vehicle (control)	6.3	6.3	9.5
SET-M33L**/**7.5	6.0	4.0	8.5
SET-M33L**/**150	6.0	NA	NA
SET-M33L**/**300	NA	5.5	8.5
SET-M33L**/**350	7.0	NA	NA
SET-M33L**/**400	NA	6.0	9.5
MMC**/**0.2	28.0	NA	NA
MMC**/**0.05	NA	NA	22.5
COL**/**0.06	20.5	NA	NA
COL**/**0.01	NA	NA	19.0
CPA**/**10	NA	17.0	NA

The table reports the mean of binucleated cells containing micronuclei after incubation with SET-M33L or positive controls at different concentrations. Lymphocytes, stimulated to divide by addition of PHA 48 hours prior to treatment, were exposed to SET-M33L for 3 hours (h) with and without exogenous metabolic activation (S9 mix), and for 20 hours (h) without S9 mix.

MMC: Mitomycin C; COL: Colchicine; CPA: Cyclophosphamide; NA: culture not analysed for micronucleus frequency.
