# Supplementary material for: In vitro and in vivo efficacy, toxicity, bio-distribution and resistance selection of a novel antibacterial drug candidate
Source: Sci Rep. 2016 May 12;6:26077. doi: 10.1038/srep26077 (PMC4864329; doi:10.1038/srep26077)
Supplement: Supplementary Information [file srep26077-s1.pdf]

In vitro and in vivo efficacy, toxicity, bio-distribution and resistance selection of a novel antibacterial drug candidate

Jlenia Brunetti,[a] Chiara Falciani,[b] Giulia Roscia,[a] Simona Pollini,[a] Stefano Bindi,[a,c] Silvia Scali,[a] Unai Cossio Arrieta,[d] Vanessa Gómez-Vallejo,[d] Leila Quercini,[a] Elisa Ibba,[a] Marco Prato,[e] Gian Maria Rossolini,[a,e,f,g] Jordi Llop,[d] Luisa Bracci,[a,c] and Alessandro Pini,[a,c,*]

[a] Department of Medical Biotechnologies, University of Siena, Siena, Italy

[b] SetLance srl, via Fiorentina 1, Siena

[c] Clinical Pathology Laboratory, Azienda Ospedaliera Universitaria Senese, Policlinico Le Scotte, viale Bracci, Siena, Italy

[d] Radiochemistry and Nuclear Imaging Group CIC biomaGUNE, San Sebastián, Spain

[e] Department of Experimental and Clinical Medicine, University of Florence, Italy

[f] Microbiology and Virology Unit, Florence Careggi University Hospital, Florence, Italy

[g] Don Carlo Gnocchi Foundation I.R.C.C.S., Florence, Italy

Table S1. MIC values for SET-M33L and for colistin as comparator antibiotic and major resistance phenotypes and genotypes of the collection of *P. aeruginosa* clinical isolates.

Sample ID	MIC SET-M33L		MIC colistin		Relevant resistance phenotype [a, b]	Relevant resistance genotype
	µg/mL	µM	µg/mL	µM		
SI_P1	8	1.4	≤0.5	≤0.4	none	
SI_P2	8	1.4	2	1.5	CARB, β/I, ESC, FQ, AG	*bla*$_{VIM}$
SI_P3	8	1.4	2	1.5	none	
SI_P4	8	1.4	≤0.5	≤0.4	CARB, β/I, ESC, FQ, AG	
SI_P5	8	1.4	≤0.5	≤0.4	CARB, ESC, β/I, FQ, AG	
SI_P6	8	1.4	2	1.5	none	
SI_P7	8	1.4	≤0.5	≤0.4	CARB, β/I, ESC, FQ, AG	
SI_P8	8	1.4	≤0.5	≤0.4	CARB, ESC, AG	
SI_P9	8	1.4	≤0.5	≤0.4	CARB, β/I, ESC, FQ, AG	
SI_P10	8	1.4	≤0.5	≤0.4	CARB, β/I, ESC, FQ, AG	
SI_P11	8	1.4	2	1.5	CARB, β/I, ESC, FQ, AG	
SI_P12	16	2.8	2	1.5	CARB, β/I, ESC, FQ	
SI_P13	8	1.4	2	1.5	none	
SI_P14	8	1.4	2	1.5	CARB	
SI_P15	8	1.4	≤0.5	≤0.4	CARB, β/I, ESC, FQ, AG	
SI_P16	8	1.4	2	1.5	CARB, β/I, ESC, FQ, AG	
SI_P17	8	1.4	≤0.5	≤0.4	CARB, β/I, ESC, FQ, AG	*bla*$_{VIM}$
SI_P18	8	1.4	1	0.7	none	
SI_P19	8	1.4	2	1.5	none	
SI_P20	8	1.4	≤0.5	≤0.4	none	
SI_P21	8	1.4	1	0.7	FQ, AG	
SI_P22	8	1.4	≤0.5	≤0.4	none	
SI_P23	8	1.4	≤0.5	≤0.4	none	
SI_P24	8	1.4	≤0.5	≤0.4	none	
SI_P25	8	1.4	≤0.5	≤0.4	AG	
SI_P26	8	1.4	≤0.5	≤0.4	none	
SI_P27	8	1.4	≤0.5	≤0.4	CARB	
SI_P28	2	0.35	≤0.5	≤0.4	none	
SI_P29	8	1.4	≤0.5	≤0.4	none	
SI_P30	8	1.4	≤0.5	≤0.4	none	
SI_P31	8	1.4	≤0.5	≤0.4	none	
SI_P32	8	1.4	≤0.5	≤0.4	none	
SI_P33	4	0.7	≤0.5	≤0.4	CARB, β/I, ESC, FQ	
SI_P34	4	0.7	≤0.5	≤0.4	CARB, β/I	
SI_P35	8	1.4	2	1.5	CARB, β/I, ESC, FQ	
SI_P36	8	1.4	≤0.5	≤0.4	CARB, AG	
SI_P37	8	1.4	≤0.5	≤0.4	CARB, β/I, ESC, FQ, AG	
SI_P38	8	1.4	≤0.5	≤0.4	CARB, β/I, ESC, FQ, AG	*bla*$_{VIM}$

SI_P39	8	1.4	≤0.5	≤0.4	CARB, β/I, ESC, FQ, AG	*bla*$_{VIM}$
SI_P40	16	2.8	≤0.5	≤0.4	none	
SI_P41	8	1.4	≤0.5	≤0.4	CARB, β/I, ESC, FQ, AG	
SI_P42	8	1.4	≤0.5	≤0.4	none	
SI_P43	8	1.4	≤0.5	≤0.4	FQ	
SI_P44	4	0.7	≤0.5	≤0.4	CARB, ESC, FQ, AG	
SI_P45	8	1.4	≤0.5	≤0.4	none	
SI_P46	8	1.4	≤0.5	≤0.4	none	
SI_P47	8	1.4	≤0.5	≤0.4	none	
SI_P48	8	1.4	≤0.5	≤0.4	none	
SI_P49	8	1.4	≤0.5	≤0.4	CARB, β/I, ESC, FQ, AG	
SI_P50	4	0.7	≤0.5	≤0.4	none	
SI_P51	8	1.4	≤0.5	≤0.4	CARB, β/I, ESC, FQ, AG	
SI_P52	8	1.4	≤0.5	≤0.4	CARB, AG	
SI_P53	8	1.4	≤0.5	≤0.4	CARB, β/I, ESC, FQ	
SI_P54	8	1.4	≤0.5	≤0.4	CARB, β/I, FQ, AG	
SI_P55	8	1.4	≤0.5	≤0.4	none	
SI_P56	8	1.4	≤0.5	≤0.4	CARB	
SI_P57	4	0.7	≤0.5	≤0.4	β/I	
SI_P58	8	1.4	≤0.5	≤0.4	FQ, AG	
SI_P59	8	1.4	≤0.5	≤0.4	β/I, ESC	
SI_P60	8	1.4	2	1.5	CARB, β/I, ESC, FQ, AG	*bla*$_{VIM}$
SI_P61	8	1.4	≤0.5	≤0.4	CARB, β/I, ESC, FQ, AG	*bla*$_{VIM}$
SI_P62	8	1.4	2	1.5	CARB, β/I, ESC, FQ, AG	*bla*$_{VIM}$
SI_P63	8	1.4	≤0.5	≤0.4	CARB, β/I, ESC, FQ, AG	*bla*$_{VIM}$
SI_P64	8	1.4	≤0.5	≤0.4	CARB, β/I, ESC, FQ, AG	
SI_P65	8	1.4	2	1.5	CARB, β/I, ESC, FQ, AG	
SI_P66	4	0.7	≤0.5	≤0.4	CARB, FQ	
SI_P67	8	1.4	≤0.5	≤0.4	CARB, β/I, ESC, FQ, AG	*bla*$_{VIM}$
SI_P68	8	1.4	≤0.5	≤0.4	none	
SI_P69	4	0.7	2	1.5	CARB	
SI_P70	4	0.7	≤0.5	≤0.4	CARB	
SI_P71	64	11.2	≤0.5	≤0.4	none	
SI_P72	8	1.4	2	1.5	FQ, AG	
SI_P73	8	1.4	≤0.5	≤0.4	β/I	
SI_P74	8	1.4	≤0.5	≤0.4	none	
SI_P75	8	1.4	2	1.5	CARB, β/I, ESC, FQ, AG	*bla*$_{IMP}$
SI_P76	8	1.4	1	0.7	CARB, β/I, ESC, FQ, AG	*bla*$_{IMP}$

[a] CARB, carbapenems; β/I, β-lactams/inhibitors combinations; ESC, extended-spectrum cephalosporins; FQ, fluoroquinolones; AG, aminoglycosides.

[b] Non susceptible to at least one agent among the following tested antibiotics: CARB, imipenem and/or meropenem; ESC, ceftazidime and/or cefepime; AG, gentamicin and/or tobramycin and/or amikacin. Resistance phenotype towards β/I and FQ was reported in case of non-susceptibility to piperacillin/tazobactam and ciprofloxacin respectively.

Table S2. MIC values for SET-M33L and for colistin as comparator antibiotic and major resistance phenotypes and genotypes of the collection of *K. pneumoniae* clinical isolates.

Sample ID	MIC SET-M33L		MIC colistin		Relevant resistance phenotype [a, b]	Relevant resistance genotype
	µg/mL	µM	µg/mL	µM		
SI_K1	16	2.8	32	24	CARB, β/I, ESC, FQ, AG, COL	*bla*$_{NDM}$
SI_K2	8	1.4	≤0.5	≤0.4	CARB, ESC, FQ, AG	*bla*$_{CTX-M}$
SI_K3	4	0.7	0.5	0.4	CARB, β/I, ESC, AG	*bla*$_{VIM}$
SI_K4	4	0.7	0.5	0.4	CARB, β/I, ESC, FQ	*bla*$_{OXA-48}$
SI_K5	4	0.7	0.5	0.4	CARB, β/I, ESC, FQ	*bla*$_{OXA-48}$
SI_K6	4	0.7	0.5	0.4	CARB, β/I, ESC, FQ, AG	*bla*$_{KPC}$
SI_K7	4	0.7	0.5	0.4	CARB, β/I, ESC, FQ	*bla*$_{KPC}$
SI_K8	4	0.7	≤0.5	≤0.4	CARB, β/I, ESC, FQ, AG	*bla*$_{KPC}$
SI_K9	8	1.4	2	1.5	CARB, β/I, ESC, FQ, AG	*bla*$_{KPC}$
SI_K10	32	5.6	>32	>24	CARB, β/I, ESC, FQ, COL	*bla*$_{KPC}$
SI_K11	16	2.8	≤0.5	≤0.4	CARB, β/I, ESC, FQ, AG	*bla*$_{KPC}$
SI_K12	8	1.4	≤0.5	≤0.4	none	
SI_K13	8	1.4	≤0.5	≤0.4	β/I, ESC, FQ, AG	ESBL [c]
SI_K14	8	1.4	≤0.5	≤0.4	none	
SI_K15	4	0.7	≤0.5	≤0.4	none	
SI_K16	4	0.7	≤0.5	≤0.4	none	
SI_K17	64	11.2	>32	>24	CARB, β/I, ESC, FQ, AG, COL	blaKPC
SI_K18	8	1.4	≤0.5	≤0.4	none	
SI_K19	8	1.4	≤0.5	≤0.4	ESC	ESBL
SI_K20	8	1.4	≤0.5	≤0.4	none	
SI_K21	4	0.7	≤0.5	≤0.4	none	
SI_K22	4	0.7	≤0.5	≤0.4	none	
SI_K23	4	0.7	≤0.5	≤0.4	none	
SI_K24	4	0.7	≤0.5	≤0.4	none	
SI_K25	8	1.4	≤0.5	≤0.4	none	
SI_K26	8	1.4	≤0.5	≤0.4	none	
SI_K27	8	1.4	≤0.5	≤0.4	none	
SI_K28	4	0.7	≤0.5	≤0.4	β/I, ESC, FQ, AG	ESBL
SI_K29	8	1.4	0.5	0.4	ESC, AG	*bla*$_{CTX-M}$
SI_K30	8	1.4	≤0.5	≤0.4	none	
SI_K31	16	2.8	16	12	β/I, ESC, FQ, AG, COL	ESBL
SI_K32	8	1.4	≤0.5	≤0.4	none	
SI_K33	8	1.4	≤0.5	≤0.4	none	
SI_K34	4	0.7	≤0.5	≤0.4	none	
SI_K35	8	1.4	≤0.5	≤0.4	CARB, β/I, ESC, FQ, AG	*bla*$_{KPC}$
SI_K36	>64	>11.2	>32	>24	CARB, β/I, ESC, FQ, AG, COL	*bla*$_{KPC}$
SI_K37	16	2.8	≤0.5	≤0.4	none	
SI_K38	32	5.6	≤0.5	≤0.4	none	
SI_K39	16	2.8	≤0.5	≤0.4	none	

SI_K40	64	11.2	>32	>24	CARB, β/I, ESC, FQ, AG, COL	*bla*$_{KPC}$
SI_K41	8	1.4	≤0.5	≤0.4	β/I, ESC, FQ, AG	ESBL
SI_K42	8	1.4	≤0.5	≤0.4	none	
SI_K43	8	1.4	≤0.5	≤0.4	none	
SI_K44	8	1.4	≤0.5	≤0.4	none	
SI_K45	8	1.4	≤0.5	≤0.4	FQ	
SI_K46	32	5.6	≤0.5	≤0.4	β/I	
SI_K47	8	1.4	≤0.5	≤0.4	none	
SI_K48	64	11.2	≤0.5	≤0.4	none	
SI_K49	8	1.4	≤0.5	≤0.4	none	
SI_K50	16	2.8	≤0.5	≤0.4	none	
SI_K51	8	1.4	≤0.5	≤0.4	none	
SI_K52	16	2.8	≤0.5	≤0.4	none	
SI_K53	8	1.4	≤0.5	≤0.4	β/I, ESC, FQ	ESBL
SI_K54	8	1.4	≤0.5	≤0.4	none	
SI_K55	8	1.4	≤0.5	≤0.4	β/I, ESC, FQ, AG	ESBL
SI_K56	4	0.7	≤0.5	≤0.4	none	
SI_K57	16	2.8	≤0.5	≤0.4	β/I, FQ	
SI_K58	16	2.8	≤0.5	≤0.4	none	
SI_K59	8	1.4	≤0.5	≤0.4	none	
SI_K60	8	1.4	≤0.5	≤0.4	none	
SI_K61	4	0.7	≤0.5	≤0.4	CARB, β/I, ESC, FQ	
SI_K62	4	0.7	≤0.5	≤0.4	CARB, β/I, ESC, FQ, AG	ESBL
SI_K63	8	1.4	≤0.5	≤0.4	β/I, ESC, FQ, AG	ESBL
SI_K64	8	1.4	≤0.5	≤0.4	none	
SI_K65	4	0.7	≤0.5	≤0.4	none	
SI_K66	4	0.7	≤0.5	≤0.4	β/I, ESC, FQ, AG	ESBL
SI_K67	4	0.7	≤0.5	≤0.4	β/I, ESC, FQ, AG	ESBL
SI_K68	4	0.7	≤0.5	≤0.4	none	
SI_K69	8	1.4	≤0.5	≤0.4	β/I, ESC, FQ, AG	ESBL
SI_K70	4	0.7	≤0.5	≤0.4	β/I, ESC, FQ	
SI_K71	16	2.8	≤0.5	≤0.4	CARB, β/I, ESC, FQ	*bla*$_{KPC}$
SI_K72	16	2.8	>32	>24	CARB, β/I, ESC, FQ, AG, COL	*bla*$_{NDM}$
SI_K73	8	1.4	≤0.5	≤0.4	CARB, ESC, FQ, AG	*bla*$_{CTX-M}$

[a] CARB, carbapenems; β/I, β-lactams/inhibitors combinations; ESC, extended-spectrum cephalosporins; FQ, fluoroquinolones; AG, aminoglycosides; COL, colistin.

[b] Non susceptible to at least one agent among the following tested antibiotics: CARB, imipenem and/or meropenem and/or ertapenem; β/I, piperacillin/tazobactam and/or amoxicillin/clavulanate; ESC, ceftazidime and/or cefotaxime and/or cefepime; AG, gentamicin and/or amikacin. Resistance phenotype towards FQ was reported in case of non-susceptibility to ciprofloxacin.

[c] ESBL production was deduced on the basis of the results of the ESBL screening test on automated system Vitek®2.

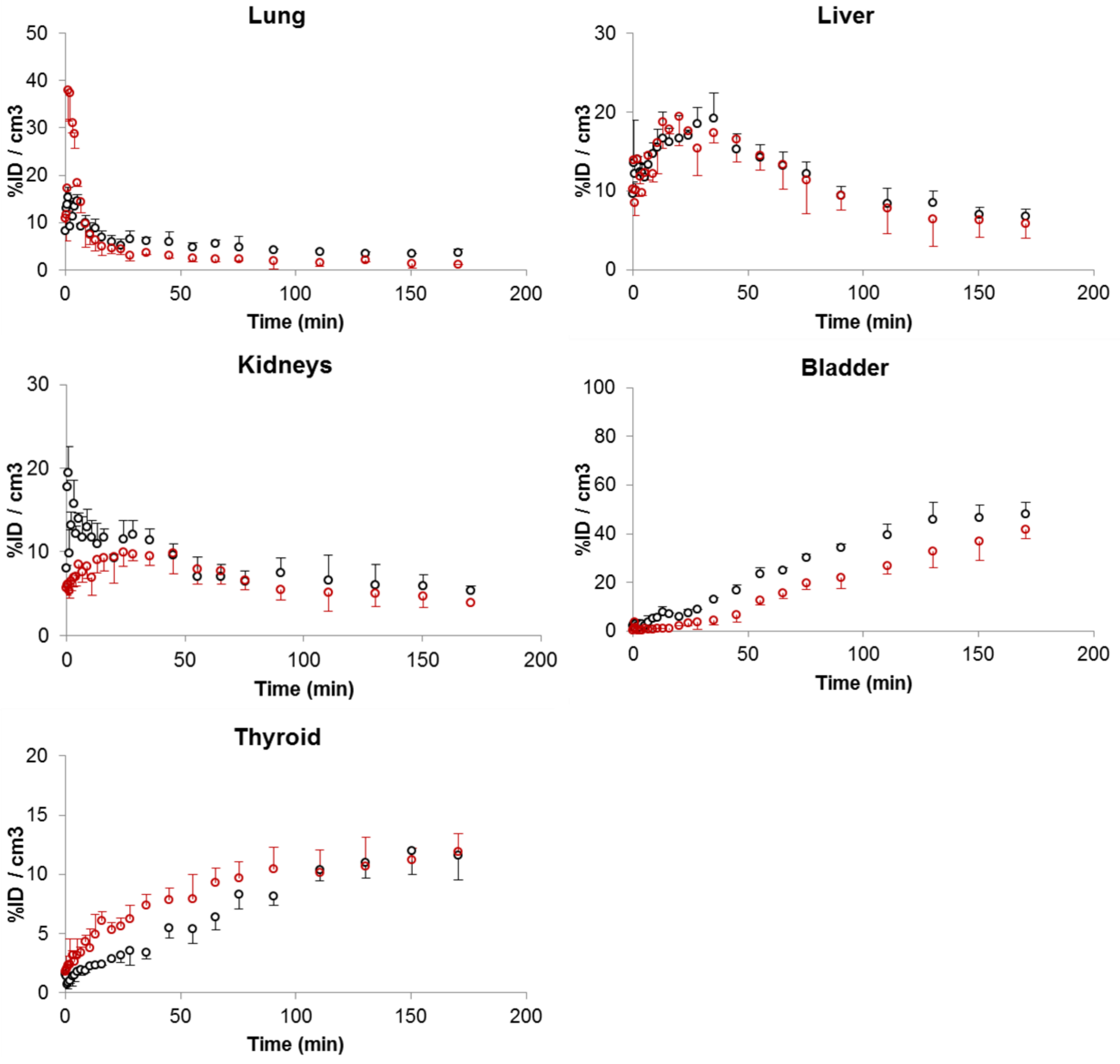

Figure S1. Accumulation of $[^{124}I]$SET-M33L (black) and $[^{124}I]$SET-M33L-PEG (red), expressed as % of injected dose per cm^3 of tissue, in different organs after intravenous administration, as determined by PET-CT.

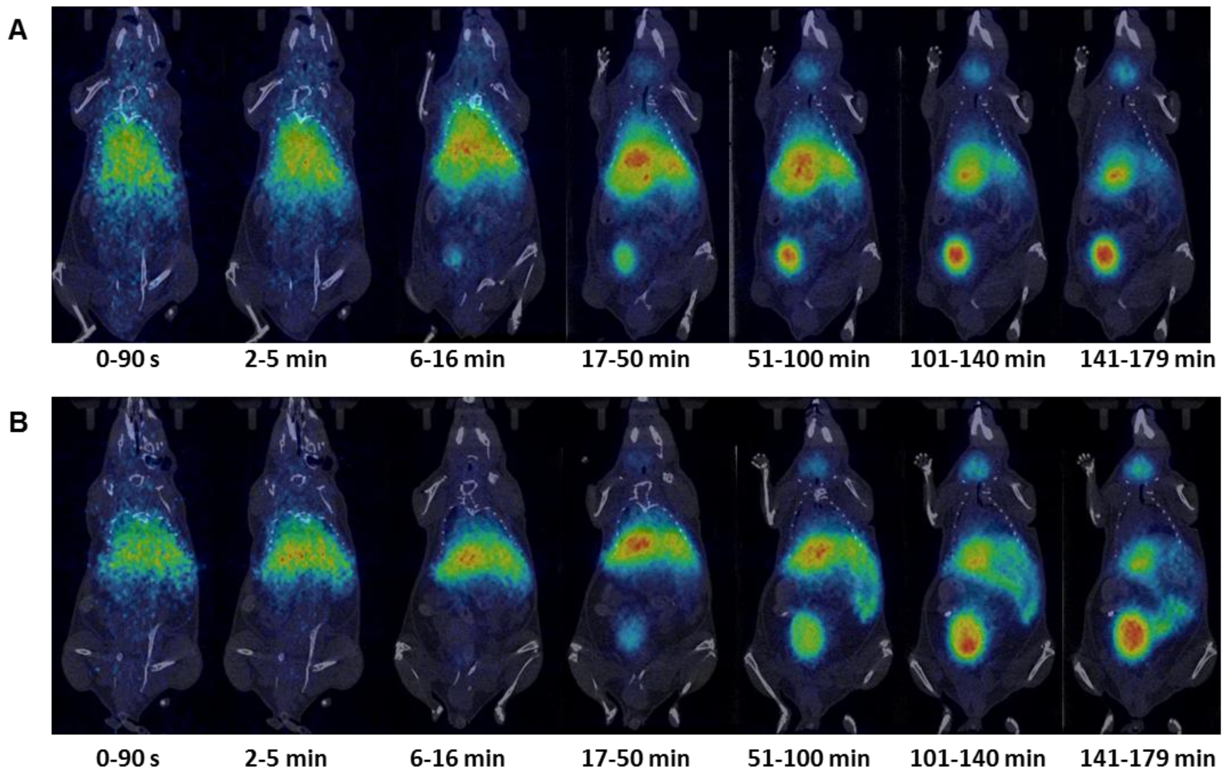

Figure S2. PET-CT images obtained at different times after intravenous administration of [^{124}I]SET-M33L (A) and [^{124}I]SET-M33L-PEG (B).
